# Supplementary material for: Mechanisms governing the pioneering and redistribution capabilities of the non-classical pioneer PU.1
Source: Nat Commun. 2020 Jan 21;11:402. doi: 10.1038/s41467-019-13960-2 (PMC6972792; doi:10.1038/s41467-019-13960-2)
Supplement: Supplementary file 7 — Source data [file 41467_2019_13960_MOESM7_ESM.zip › Source_Data/FigureS2/FigureS2B_MotifScanOutput/KmeansCluster5/homerResults/motif3.similar.html]

motif3

## Information for motif3

A
T
C
G
C
T
G
A
A
C
T
G
C
G
T
A
C
G
T
A
A
C
T
G
A
C
G
T
C
T
A
G
  
Reverse Opposite:  

A
G
T
C
C
G
T
A
A
G
T
C
A
C
G
T
A
C
G
T
A
G
T
C
A
G
C
T
A
T
G
C
  

|  |  |
| --- | --- |
| p-value: | 1e-344 |
| log p-value: | -7.931e+02 |
| Information Content per bp: | 1.914 |
| Number of Target Sequences with motif | 1107.0 |
| Percentage of Target Sequences with motif | 27.81% |
| Number of Background Sequences with motif | 2637.9 |
| Percentage of Background Sequences with motif | 6.16% |
| Average Position of motif in Targets | 102.5 +/- 44.5bp |
| Average Position of motif in Background | 100.4 +/- 77.6bp |
| Strand Bias (log2 ratio + to - strand density) | 0.2 |
| Multiplicity (# of sites on avg that occur together) | 1.07 |
| Motif File: | file (matrix) reverse opposite |

### Similar de novo motifs found

|  |  |  |  |  |  |  |  |
| --- | --- | --- | --- | --- | --- | --- | --- |
| Rank | Match Score | Redundant Motif | P-value | log P-value | % of Targets | % of Background | Motif file |
| 1 | 0.757 | C T A G C A T G C T A G C T A G T C G A C T G A T C A G C A G T A C T G T A G C G A C T C G T A | 1e-177 | -407.774610 | 7.64% | 0.57% | motif file (matrix) |
| 2 | 0.752 | T G A C G C T A T A G C G C A T C A G T G T A C G C A T T C A G G A T C G C T A G C A T | 1e-95 | -218.779444 | 7.81% | 1.61% | motif file (matrix) |
